# Supplementary material for: Are carbon emissions trading and green financial instruments synergistic? -Comprehensive quantitative research based on content analysis
Source: PLoS One. 2024 Mar 7;19(3):e0298601. doi: 10.1371/journal.pone.0298601 (PMC10919601; doi:10.1371/journal.pone.0298601)
Supplement: S1 Data — (ZIP) [file pone.0298601.s001.zip › Data/地级市Z_F工作报告.zip!/╕╩╦α/╒┼╥┤╩╨/└┤╫╘╣1⁄2╓┌║┼í╛┬φ┐╦╩2╛▌═°í┐_2018.docx]

各位代表：

现在，我代表市人民政府向大会报告工作，请予审议，并请各位政协委员和列席人员提出意见。

2017 年政府工作回顾

刚刚过去的一年 ，在省委、省政府和市委的坚强领导下，我们深入贯彻党的十八大、十九大精神，全面落实习近平总书记视察甘肃重要讲话和“八个着力”重要指示精神，统筹推进稳增长、促改革、调结构、惠民生、防风险、保生态各项工作，经济总量稳中有进，产业结构更趋优化，民生保障持续加强，生态建设有力有序，社会大局和谐稳定。全面建成小康社会实现程度综合指数达88.8%，位居全省第5。预计全年实现生产总值406亿元，增长3%。完成固定资产投资245亿元，下降30%。实现社会消费品零售总额174亿元，增长8.3%。完成一般公共预算收入26.32亿元，增长1.48%。城镇、农村居民人均可支配收入可达23223元和12578元，均增长8%。存款余额614.45亿元，同比增长5.2%；存贷比达99.14%，提高4个百分点。

（一）全面加强整治保护修复，生态文明建设成效明显。 积极践行绿水青山就是金山银山的理念，深刻汲取祁连山生态环境问题的教训，以贯彻中央《通报》精神、狠抓突出问题整改为重中之重，推动形成绿色发展方式和生活方式，生态文明建设持续加强。

祁连山生态环境问题整治成效显著。 坚决贯彻落实习近平总书记关于祁连山生态环境保护重要批示精神及中央、省市委决策部署和市人大常委会决议，制定出台9个专项整治方案和3个方面的整改意见，层层传导压力，集中力量攻坚，全面推进祁连山生态环境突出问题整改整治。由我市负责整改的171项问题，已全面完成现场整治任务170项、正在整改1项，保护区范围内矿山探采项目全面关停，开发性生产经营活动全面停止，水电站生态基流下泄监控实现全覆盖，核心区149户农牧民搬迁工作全面完成，整治工作取得阶段性成效。《祁连山国家公园体制试点方案》获得国家批复，祁连山生态系统重建有序推进。

黑河湿地生态保护治理扎实推进。 坚决关停黑河湿地自然保护区核心区、缓冲区内所有生产经营项目，有序退出实验区内经营项目。按照逐点核实、整改销号的要求，全面整改环保部卫星遥感监测反馈问题，已完成整改257项、正在整改2项。湿地湖泊生态环境保护项目完成投资1.14亿元，总投资2.2亿元的湿地保护与治理、转移转产项目顺利实施，湿地保护区范围和功能区划调整工作取得阶段性成果。

山水林田湖草生态工程加快实施。 全面启动实施祁连山、黑河湿地国家级自然保护区“两转四退四增强”行动计划，成功申报获批总投资52.6亿元的山水林田湖草生态保护修复项目，到位中央、省级支持资金17.8亿元。矿山环境治理恢复、黑河沿岸防护林、黑臭水体治理等55项工程加快实施，完成实物投资量12.3亿元。持续推进大规模国土绿化，完成营造林22万亩。

全域生态环境整治持续加强。 实行最严格大气污染防治管理，加大扬尘管控，拆除燃煤锅炉134台，治理油烟污染餐饮经营单位2300多家，改炕5500多户，淘汰报废黄标车及老旧车2100多辆，空气质量持续改善，名列全省前茅。全面推行“河长制”，查处52家违法违规排放企业， 实施 水污染防治工程21项，饮用水水源地保护进一步加强。 黑河城区段治理累计完成投资4亿元。中央财政投资 1.3 亿元的民乐铬盐厂铬污染土壤修复项目加紧实施。启动全域无垃圾行动，探索建立废旧地膜回收处置机制，农村垃圾收集体系初步建立，城乡环境面貌持续改观。

（二）着力彰显禀赋优势，产业特色更加鲜明。 紧紧围绕转方式、扩规模、强质量、提效益，持续培育壮大主导产业，发展优势不断显现。

现代农业稳步发展。 大力发展优势主导产业和区域特色产业， “ 四个百万”工程扎实推进。新增蔬菜5万亩、食用菌3万吨，新改扩建标准化规模养殖场区102个，农业产业结构进一步优化。三元乳业等35个重点加工项目建成投产，绿洲5万吨恒温保鲜库等22项投资千万元以上项目加快建设，完成投资23亿元，农产品加工转化率达到62%，产业化水平持续提高。海升集团现代智能温室工业化栽培生态示范和7个戈壁农业示范园区项目落地实施，非耕地设施农业发展取得新突破。建成绿色有机农产品示范基地64个，新认证有机农产品8个、绿色食品16个，建成质量安全追溯点58处，高台国家农业可持续发展试验区获批创建，甘州、民乐被确定为全省首批有机产品认证示范区，农业标准化、安全化体系不断完善。

工业经济止滑企稳。 深入落实“三一”“三重”工作部署，着力促进企业提质增效、产业不断壮大。持续推进工业园区基础设施建设，完成投资4.7亿元，园区功能更趋完善、聚集带动作用显著提升。开工建设投资千万元以上重点工业项目115项，滨河酒文化产业园、常大钢结构装配式建筑等58项重点项目建成投产，晋昌源煤业20万吨中温焦油加氢处理、鼎丰源凹凸棒高效综合开发等重点项目加快建设，完成投资45.3亿元。大力支持企业创新发展，实施金南瓜奶油南瓜粉等技术创新项目28项，完成投资7.8亿元。积极扶持企业降低成本、扩大生产，为实体企业补贴电价3350万元，支持应急周转金7.4亿元，担保融资11.2亿元。全面实行领导联系帮扶制度，帮助企业解决困难问题，有效促进了工业经济止滑企稳。

旅游产业持续升温。 全力推进旅游文化体育医养融合和全域旅游发展战略，着力深化“四大行动”，旅游业继续保持强劲发展势头。丹霞景区　5A　级创建工作具备验收条件。 新建续建滨河新区自驾游基地、丝路江南生态古镇等旅游项目79 项，完成投资32亿元； 七彩丹霞直升机、动力伞等一批体验性项目投入运营，新培育乡村旅游示范点8个；新建改建旅游厕所128座，新增旅游接待床位4000多张；成功举办中国汽车拉力锦标赛、青少年帆船城市邀请赛等22项大型赛事，户外运动品牌影响力不断扩大， 旅游延链补链成效明 显。持续强化旅游市场秩序整治，查处旅游投诉63起；举办旅游培训班61期1万多人次，旅游管理服务能力持续提高。举办大中型城市旅游推介营销活动12场次，开通旅游专列300趟、直航包机122班，央视《魅力中国城》竞演活动影响力巨大，张掖旅游知名度不断提升。 全年接待游客2599万人次，实现旅游综合收入157亿元，分别 增长28%和37.7% 。

（三）全力加快项目建设，发展基础更趋稳固 。 全面落实项目建设“六个清单”工作任务和“五个一”工作机制，开工建设各类项目624项， 固定资产投资降幅逐月收窄，增速 位 居全省第5。

城乡建设力度加大。 实施各类城建项目292项，完成投资79.5亿元，其中市政基础设施建设投资达23.9亿元，超过前两年总和。修编完成老城区、滨河新区等控制性详细规划及主城区地下管廊、海绵城市等一批专项规划。新改拓建城市道路41条　48公里　，改造背街小巷8条，完成市区南二环、新建街、长寿街等道路精品化改造工程。市污水处理厂一期提标技改、临泽县城区污水处理厂再生水利用等工程建成投用，配套建设城市污水收集管网　143公里　，市区三水厂扩建、肃南县城备用水源等供水工程加快建设。市区热电联产集中供热扩容及高台、山丹、民乐县城区集中供热工程全面建成，新增集中供热面积920万平方米。新增天然气用户1.25万户。华西能源 垃圾焚烧发电一期 建成投运，甘州、临泽、山丹、民乐城乡生活垃圾转运处理一体化项目启动实施。大力推进“清雅甘州”改造，完成190栋65万平方米沿街既有建筑节能及色彩改造任务。启动城市违法建筑治理三年行动，查处违法建筑26.　9万平方米　。实施各类村镇基础设施项目624项，完成投资10.5亿元。扎实推进69个“千村美丽”示范村和95个“万村整洁”村建设，完成投资8.2亿元。

基础设施不断完善。 完成交通基础建设投资40亿元，新改建农村公路　420公里　。滨河新区黑河大桥、临泽沙柳公路及山丹大佛寺旅游公路建成通车，S237甘平一级公路全线贯通。G312临泽过境段、G227六东一级公路二期、S590山丹至马场等12项国省干线加快建设，张掖客运中心站等重点站场项目有序推进。完成水利投资8.5亿元，民乐山城河、山丹白石崖水库开工建设，大型灌区节水改造、农村饮水安全、山洪灾害防治等重点工程进展顺利。永久基本农田划定工作全面完成，整理高标准农田44.4万亩。龙首110千伏、顺化330千伏变电站等工程建成投用。“宽带中国”示范城市和电信普遍服务试点城市创建任务全面完成，智慧城市建设获得省级PPP引导资金2700万元，城市大数据中心建成投用，7个子项目建设取得突破性 进展。

招商引资持续加强。 新签约招商项目205项，总投资368.4亿元。新建续建招商项目351项，到位资金296.2亿元，其中省外288.9亿元。成功举办“陇商回家乡·再聚金张掖”、中国·张掖陇药博览会等大型招商引资节会。市县两级筛选储备PPP项目624项，其中44项进入国家级项目库、68项进入省级项目库，总投资60亿元的张扁高速入选国家第三批PPP示范项目并开工建设，市区热电联产项目被列为国家存量资产盘活PPP模式示范项目，张掖被列为全国市政工程领域PPP创新工作重点城市。

（四）强力推进改革创新，内生动力持续增强。 坚持把改革、创新、开放作为增强发展活力的主抓手，全面推进重点领域和关键环节改革，发展环境持续优化，发展活力动力不断增强。

“双创示范”任务全面完成。 修订完善“黄金19条”、双创资金管理办法等8项扶持政策措施，奖励补助科技孵化器、双创基地、创新型企业2410万元，培育双创典型106个，发放“助创贷”“助保贷”“互助贷”11.94亿元、扶持基金1.5亿元，贴息担保贷款8.35亿元，政策吸引聚集驱动效应更加有效。新增国家小微型企业双创示范基地1家、众创空间2家，省级创业就业孵化示范基地2家、科技孵化器3家。“双创城市示范”创建以来，全市共建成创业创新孵化园、科技孵化器、众创空间、公共服务示范平台103家，入驻入孵企业2600多户，带动就业4.9万人；新增小微企业1.53万户，小微企业拥有授权专利1530件；全市新增城镇就业9.57万人，小微企业实现就业5.3万人、营业收入1169亿元、上缴税金45亿元；新增高新技术企业53家，技术合同交易额达49.5亿元，撬动社会资本投入56亿元。全面超额完成各项创建目标任务。

“放管服”改革不断深化。 集中公布行政审批中介服务事项、工商登记前置审批事项目录等“六张清单”，新取消调整审批事项8项。依据法律法规立改废，及时调整部门单位权责清单38项。市政务服务数据交换平台建成投用，行政许可事项实现网上申报办理。优化流程，加强监督，公共资源交易市场秩序明显好转。加快推进商事制度改革，办理“多证合一”营业执照1000多户，160户企业通过简易程序办理注销登记。全面推行“双随机一公开”监管模式，事中事后监管进一步规范。持续深化环评审批制度改革，环评效率进一步提高、成本进一步降低、监管进一步加强。

重点领域改革稳步推进。 农村土地确权工作基本完成，土地流转交易额达2.5亿元、产权交易贷款达15.2亿元，“三权分置”工作稳步推进，高台县被列为全国集体资产股份合作制改革试点县。 完成首例排污许可证有偿使用交易试点。 五大投融资平台融资15.4亿元，完成投资29.2亿元，为企业担保贷款6.3亿元，撬动社会资本、放大投资效应进一步显现。积极推进普惠金融机制改革，金融支持小微企业、农民和特殊群体发展的力度进一步加大。鸿泰种业在“新三板”挂牌，31户企业纳入“新三板”培育库，332户企业在甘肃股权交易中心挂牌，2户企业在资本市场直接融资4000万元。

对外开放持续扩大。 出台落实 外贸服务平台招引、人才招聘、专项应急周转金设立等集成配套政策，外贸经济加快发展 ，实现自营出口2亿元，增长26%。“金张掖夏菜”等特色农产品外销量持续增长，农业“东进西出”取得新突破。建成运营电商交易平台20多家，新发展电商企业200多家，农产品外销额达7.7亿元，增长30%。着力完善商流市场体系，愿景商贸城二期、山丹世博丽景商业广场等21项城市商流基础设施项目加快建设，完成投资14.1亿元。成功举办第四届“张交会”，签订购销合同7.5亿元，多重效益和对外影响力持续提升。

（五）着力促进社会和谐，民生保障不断加强。 坚持把提升人民群众生活质量作为发展的根本取向，大力发展社会各项事业，着力完善社会保障体系，公共服务惠民水平持续提升。

社会事业全面协调发展。 坚持教育优先发展战略 ，建成滨河新区高中，甘州和民乐思源实验学校等重点项目加快建设，民乐县通过义务教育均衡发展国家评估认定，高考录取率稳居全省首位。 新建、 改扩建幼儿 园60所，学前教育入园率达99.1% ， 城区“入园难”问题得到有效缓解。持续推进医药卫生体制改革，市第二人民医院建成主体工程，公立医疗卫生机构基本药物零差率销售实现全覆盖，基本公共卫生服务水平不断提高。全面两孩政策稳步推进，28万人次计生家庭享受人口优惠政策补助9430万元。实施各类科技项目33项，科技对经济增长的贡献率达到52%。深化国家公共文化服务体系示范区创建，市图书馆、博物馆、美术馆、数字图书馆建成投用，市文化馆和临泽城市文化综合体、民乐“五馆”、山丹“七馆”加快建设，新建社区综合性文化服务中心17个、“乡村舞台”100个、博物馆15个，西来寺、土塔等16个重点文物保护项目顺利实施，国家扩大城乡居民文化消费试点城市创建工作深入推进。实施各类体育基础设施惠民工程38项，全民健身及群众性体育活动蓬勃开展。

社会保障体系不断完善。 落实各项扶持政策，全力稳定和扩大就业，城镇新增就业2.52万人，城镇登记失业率控制在2.6%以内。着力扩大社会保险覆盖面，城乡居民基本养老保险续保率达95%。跨省异地就医实现直接结算，城乡居民医保整合顺利完成。 实施棚户区改造18220套（户），发放住房租赁补贴6190户1367万元。 城、乡低保和特困供养标准分别提高8%、22.6%和8.4%，医疗救助、临时救助、孤残保障政策全面落实。新改扩建高标准敬老院7所、社区日间照料中心80个，甘州全国综合养老示范基地、市儿童福利院、市民政精神病福利院和临泽、甘州、山丹殡仪服务中心等一批民生保障设施建成投用。

精准脱贫成果巩固提升。 争取专项扶贫资金1.3亿元，市县两级安排专项扶贫资金1.5亿元，全部用于支持贫困村生产发展和基础设施建设。完善贷款贴息等扶持政策，大力培育特色扶贫产业，65个贫困村新建日光温室200座、养殖小区83个，新发展食用菌6000多亩，特色产业订单面积占耕地的70%以上。全力实施易地扶贫搬迁项目，开工建设安置点23个、安置房2587套。全市脱贫3378人，贫困发生率下降到1.54%。

社会大局保持和谐稳定。 强化安全生产责任，开展隐患排查整治，打击非法违法行为，安全生产四项指标大幅下降，顺利通过国家安全生产巡查和综合督查。全面落实“四个最严”和“四有两责”要求，有效保障了群众食药安全。市县救灾物资储备库加快建设，应急管理和防灾减灾救灾能力持续提高。全面贯彻党的民族宗教政策，民族团结、宗教和顺局面进一步巩固和发展。深入开展双拥共建活动，市军供站、军休站建成投用。“七五”普法扎实推进，全社会法治意识不断提升。深入推进非法集资、诈骗等金融违法活动集中整治，持续开展信访秩序综合整治，全力排处矛盾纠纷和群体性事件，全面加强禁毒、打黑和案件侦破工作，立体化信息化社会治安防控体系建设扎实有效，社会大局和谐稳定。 外事侨务、机构编制、审计统计、粮食供销、物价质监、老龄妇幼、人防气象、史志档案等工作都取得了新的进步。

（六）持续提升服务效能，政府自身建设有力有效。 扎实推进“两学一做”学习 教育常态化制度化，政府系统党建不断加强。严格执行政府决策议事规则和工作程序，严格落实 公众参与、专家论证、风险评估、合法性审查、集体讨论决定等重大行政决策制度 ，决策更加科学民主。主动接受人大法律监督和工作监督，自觉接受政协民主监督、职能机关专门监督、社会公众监督和新闻舆论监督，办结人大代表建议67件、政协提案136件，办结率100%。推进财政预决算公开，资金使用高效安全。研究制定《市政府党组落实党风廉政建 设主体责任实施办法》，严格履行“一岗双责”，认真开展“三纠三促”专项行动， 严查 “四风”问题 ，廉洁政府、高效政府建设取得新成效。

各位代表！2017年，在经济发展大环境复杂多变、祁连山生态环境问题整治任务艰巨繁重的形势下，全市上下正视困难、积极作为，经济发展实现稳定向好，民生持续改善，社会大局保持和谐稳定，生态保护、城市建设、全域旅游、创业创新等领域取得长足进步。这些成绩的取得，实属不易。在此，我代表市人民政府，向全市各族人民，向奋战在各条战线上的人大代表、政协委员、干部群众，向各民主党派、人民团体、中央和省属驻张单位，向离退休老同志、驻张部队、武警官兵、公安干警表示衷心的感谢并致以崇高的敬意！

我们也更加清醒地看到，经济社会发展和政府工作中还存在不少困难和问题。主要表现在：一是经济运行遇到前所未有的困难，部分指标没有达到年初预期，经济回稳向好的基础还很不牢固。 二是 产业性重大项目开工不足、接续不足，民间投资活力下降， 经济结构调整、产业转型升级、发展提质增效的任务还很艰巨。三是 工业投资持续下滑，停产停工企业数量较多，工业经济面临最为严峻的形势。四是祁连山生态保护修复治理还面临项目退出补偿未落实、矛盾交织叠加、监管手段落后等困难和问题，保护修复任重道远。五是市场发育度不高，区域创新力不强，要素聚集吸附能力弱，就业的稳定性和质量不高，城镇居民可支配收入水平依然较低，农村居民持续稳定增收难度加大 。六是一些部门和干部不愿担当、不敢担当、不会担当的问题比较突出，主动作为、尽力而为、奋发有为与人民群众的期待仍有很大差距，政府职能转变和效能建设仍需大力加强。这些问题，我们将高度重视并在今后工作中努力加以解决。

关于2018年政府工作

党的十九大对新时代推进中国特色社会主义伟大事业作出了全面部署，极大地鼓舞了全国人民为实现中华民族伟大复兴的中国梦而奋斗的信心和力量，为我们开启新征程、续写新篇章指明了前进方向、提供了行动纲领。我们要 把学习宣传贯彻党的十九大精神作为当前和今后一个时期政府工作的首要政治任务，深学细悟、学以致用，用党的十九大精神指引前进方向、提振必胜信心、凝聚奋进力量。

贯彻落实十九大精神，必须深刻领会新思想精髓，指导张掖发展新实践。 习近平新时代中国特色社会主义思想是马克思主义中国化的最新成果，是全党全国人民为实现中华民族伟大复兴而奋斗的行动指南。我们一定要深入学习，深刻领会新思想的丰富内涵，把“五位一体”总体布局、“四个全面”战略布局贯穿于政府工作的全领域，并通过认认真真抓落实使之在张掖得以全面贯彻；深刻领会新思想的精髓要义，把坚持以人民为中心、坚持人民当家作主、坚持人与自然和谐共处、坚持在发展中保障和改善民生等要求转化为政府为民谋利、为民造福的决策、政策、项目，使广大人民群众每年都感受到发展新变化，共享到发展新成果；深刻领会新思想举旗定向而又现实切近的指导地位，牢固树立“四个意识”，不断增强“四个自信”，坚决维护以习近平同志为核心的党中央权威和集中统一领导，使政府的各项决策、各项部署、各项工作始终与党中央保持一致，始终沿着正确的方向前进。

贯彻落实十九大精神，必须准确把握新时代特征，描绘张掖发展新蓝图。 中国特色社会主义进入了新时代，这是十九大对我国所处历史方位作出的科学判断，张掖也必然而且必须与全国一同迈入新时代。我们要准确把握夺取中国特色社会主义伟大胜利、建设社会主义现代化强国、实现全体人民共同富裕和中华民族伟大复兴中国梦的新时代伟大目标和神圣使命，把张掖发展放在新时代的大背景下去谋划，使张掖的发展适应新时代的要求、体现新时代的特色。我们要充分认识人民日益增长的美好生活需要和不平衡不充分发展之间的矛盾这一新时代特征在张掖的表现，着力提升经济发展整体水平，着力提升发展的质量和效益，着力加快农村贫困人口精准脱贫步伐，着力提升城乡低收入人群的社会保障水平，使区域、民族、城乡等发展不平衡的问题持续解决，使生态保护、城乡环境、公共服务、科技教育、医疗卫生、健康养老等发展不充分的短板得到弥补，在建设幸福美好金张掖的征程上年年有新作为、新气象。

贯彻落实十九大精神，必须全面落实新理念要求，开创张掖发展新局面。 党的十九大把贯彻新发展理念、建设现代化经济体系作为决胜全面建成小康社会、实现中华民族“两个一百年”奋斗目标的大战略，提出了要求、作出了部署，张掖的发展必须贯彻新理念、落实新要求、开创新局面。围绕做大做强优势主导产业，深化供给侧结构性改革，使张掖的产业体系更加突出市场导向、更具市场活力和竞争力；深入实施创新驱动发展战略，在人才引进、机制体制、经营管理、投资融资、科学技术等各个领域加大创新支持力度，形成一批创新成果；抓住国家实施乡村振兴战略的历史机遇，充分发挥张掖农村在资源禀赋、产业基础、经营机制、区位交通、劳动力素质等方面的比较优势，以打造绿色有机农产品生产基地为目标、以产权制度改革为突破口、以一二三产融合发展为动力，率先把张掖农村建成产业兴旺、生态宜居、乡风文明、治理有效、生活富裕的美丽乡村，开创以城带乡、以乡促城、城乡融合发展的新局面。

2018年，是贯彻党的十九大精神的开局之年，是决胜全面建成小康社会、实施“十三五”规划承上启下的关键一年。政府工作的总体要求是：全面贯彻党的十九大精神，以习近平新时代中国特色社会主义思想为指导，深入贯彻习近平总书记 视察甘肃重要讲话和 “八个着力”重要指示精神，坚持稳中求进工作总基调，坚持新发展理念，坚持以供给侧结构性改革为主线，以改革创新为动力，按照高质量发展的要求，全面做好稳增长、促改 革、调结构、惠民生、防风险、 保生态 各项工作， 促进 经济平稳健康发展和社会和谐稳定。

经济社会发展主要预期目标是：生产总值增长6%。固定资产投资增长10%。社会消费品零售总额增长8%。一般公共预算收入增长5%。城镇、农村居民人均可支配收入均增长8%。单位生产总值能耗、主要污染物排放等约束性指标控制在省上下达指标以内。

重点抓好六个方面的工作：

（一）坚定不移抓发展，力促经济持续稳定增长

坚持把抓发展作为解决一切问题的根本和关键，把贯彻五大发展理念作为抓发展的根本遵循，凝聚发展共识，提振发展信心，着力转变发展方式，全力促进高质量发展。

把握稳中求进总基调，保持经济平稳较快增长。 牢牢抓住“一带一路”建设战略机遇，准确把握稳中求进工作总基调，积极应对各类挑战，着力巩固“稳”的基础，持续积蓄“进”的力量，努力推动经济总量持续扩大、结构更加优化、质量效益不断提升。坚持以供给侧结构性改革为主线，调整存量与优化增量并重、做强实体经济与补齐发展短板并重， 在主导产业发展上抓深显特，在基础设施和重大项目建设上抓实见效，在全面建成小康社会各项指标上争先进位 。 坚持把县域经济作为转变经济发展方式的主战场，出台支持县域经济发展的政策，强权扩权打造发展平台，激活要素壮大支柱产业，优化营商环境促进民营经济发展，全面提升县域经济发展活力。坚持问题导向、底线思维，把中央防范化解重大风险、精准脱贫、污染防治三大攻坚任务全面落到实处，使发展的基础更为稳固、动力更为强劲。

实施项目带动战略，提升经济综合实力。 紧盯国家产业政策和投资趋向，瞄准生态保护、基础设施、产业培育和社会事业等重点领域，积极谋划、储备一批大项目、好项目。强化部门工作责任，加大项目、资金争取力度，力争更多项目跻进国家和省上计划盘子，祁连山和黑河湿地生态保护与建设、兰州至张掖铁路三四线等事关长远发展的重大项目及早落地实施。坚持实行重点项目“五个一”推进机制，实施好张掖通航产业园一期、花草滩循环经济产业园等重大项目，力争全年开工建设各类项目520项以上，完成固定资产投资270亿元以上。 创新PPP融资模式， 用好省上丝路 交通发展基金、PPP发展基金和“两个1000亿”专项贷款政策，扩大有效投资。推动智慧城市、奥体中心等PPP示范项目规范运作，力争更多PPP项目落地实施。

加快基础设施建设，改善发展支撑条件。 积极推进“双修”城市试点工作，按照旅游城市的定位和标准，以道路、管网、水电热气供应、学校、医院等公共服务设施为重点，着力补齐城市基础设施短板。加快创建国家园林城市，完成市区黑臭水体治理任务，开展城市公厕配套行动 ，实施市区集中供热备用热源、甘州污水处理厂尾水自然生物净化利用、民乐和临泽城区水源地建设等项目， 持续改善人居环 境。 实施县区出城口改造提升工程， 加快市区西二环、仁宗路、青年东街、民主东街、滨河新区民乐路等道路和主干道人行道改造，改造背街小巷4条，持续推进各县城区道路建设，不断提升城市综合服务功能。按照乡村振兴战略的要求，全面提升农村基础设施水平，用足用好农村人居环境整治千亿元贷款政策，持续推进12个国家级重点镇、35个建制镇、1个特色小镇建设，新建“千村美丽”示范村50个以上、“万村整洁”村80个以上。加快张扁高速、国道312张掖至临泽 一级公路建设进度，力争开工建设张掖至康乐一级公路、S301北部大通道等5项道路工程，建成六东路二期、月牙湖景区旅游公路等8条续建道路，力争开工建设山丹新城区客运站等运输站场，抓紧开展张掖城区至丹霞快速通道论证及前期工作，完成交通运输投资40亿元以上。 大力推进水利基础设施建设，实施灌区续建配套与节水改造、中小河流治理等项目，全面完成山城河、白石崖水库建设任务，开工建设一批骨干水源工程，解决2.5万人饮水安全不稳定问题，新增高效节水灌溉面积15万亩，完成水利投资10亿元以上。 加快建设750千伏送变电和330千伏甘州变项目，推进绿洲、海潮110千伏输变电等电网项目建设。加快“数字张掖”建设，新建移动通信基站900个，实现全市光网和　4G　网络全覆盖，完成投资6.5亿元。

全面扩大开放招商，激发经济发展活力。 进一步放大“双创”效应，强化专业招商、以商招商、会展招商、网络招商，积极引进行业龙头项目,扩大有效投资，提高民间投资和实体经济投资比重。 加快 红星美凯龙商业综合体、丝绸之路文化遗产博览城、三元乳业生态观光园等重点项目建设，做好万达广场等重点招商项目衔接跟进，力争早日开工建设。强化 跟踪落地服务实效， 促进签约项目加快实施、在建项目尽快投产、投产项目达产达标 。 以 培育出口基地、龙头企业和扩大出口为重点，进一步 落实支持外贸发展政策措施，加强冷链物流体系建设，支持企业在霍尔果斯、广西凭祥口岸建立办事处，打通优质农产品东进西出通道，争取大宗农产品基本实现自营出口。坚持国际化、专业化、产业化和市场化的基本定位，持续提升“张交会”品牌形象和带动效应。

（二）聚焦转型抓产业，提高发展质量效益

全面贯彻新发展理念，聚焦产业转型升级，深化供给侧结构性改革，破解资源环境瓶颈约束，加快构建资源消耗少、创新能力强、产出效益高的新型产业体系。

突出绿色取向，做优现代农业。 坚持把绿色有机农产品生产作为现代农业发展的主攻方向，以“三元双向”循环为基本模式，以现代农业园区和产业化项目为示范引领，以节约资源和提高效益为核心关键，着力构建现代农业产业体系、生产体系、经营体系，加快转型升级步伐。深入实施“四个百万”工程，持续优化产业结构，玉米制种面积稳定在100万亩左右，蔬菜产业面积扩大到80万亩；支持食用菌关键技术研究和成果转化，食用菌产量达到8万吨；新建标准化规模养殖场100个以上，新创建省部级示范场6个，人工种草面积达到138万亩。把戈壁农业作为转变农业发展方式和提升产业化水平的引擎，坚持“生态优先、规划先行、定位高端”的基本思路，加快建设海升现代智能温室工业化栽培生态示范项目，开工建设甘州明永滩、高台青草湖等9个千亩戈壁农业示范园，新发展戈壁农业2.7万亩。全力抓好黑河水电万头奶牛养殖场、祁连葡萄酒业产业融合发展、祥云5万吨啤酒麦芽生产等重点农业产业化项目建设，完成投资18亿元以上，新增农产品加工能力4万吨。强化农产品质量安全监管，大力推广标准化生产技术，着力打造张掖农产品品牌，力争新发展120家示范性专业合作社、75家示范性家庭农场，新认证“三品一标”农产品20个以上。输转劳动力28万人，实现劳务收入52亿元以上。

坚持稳中突破，做大生态工业。 持续强化“2+4”工业园区支撑体系，推行差别化定位和错位发展，进一步完善基础设施、延伸公共服务，不断提升园区聚集功能，着力提高集约化发展水平。加快在建重大工业项目建设进度，建成豫中明达2000吨间苯二酚等50个工业项目，开工建设河西制药公司二期中药材深加工、宏鑫矿业70万吨锰铁选矿扩建等60个新建项目，完成工业固定资产投资55亿元以上。全力抓好新恒基农作物秸秆循环转化利用产业园、西域蓝天太阳能新材料科技产业园等投资上亿元重点工业项目招商和落地，力争尽早开工。支持高台盐硝化工、临泽凹凸棒产业加快发展。强化市级领导联系帮扶重点财源企业和项目制度的落实执行，“一企一策”精准施策，着力解决突出问题。 继续落实企业降成本扶持政策，支持企业满负荷生产， 新增入规企业20户以上。

深化“四大行动”，做强旅游文化产业。 坚持以全域旅游为引领，全力推进旅游文化体育医养融合发展战略，加快创建国家全域旅游示范区，着力构建完整的产业链和复合型产业集群。确保 丹霞景区晋级国家　5A　级旅游景区，加快申报张掖丹霞世界自然遗产，启动申报世界地质公园。 积极推进 大佛寺、平山湖大峡谷等核心景区资源整合，深化经营管理机制改革。 大力推进重点旅游文化项目建设， 实施丹霞轨道交通、 芦水湾生态旅游区 、平山 湖喀尔喀小镇、金山 公园、屋兰古镇民俗创新示范区等旅游延链补链项目50项以上，完成投资40亿元以上。精心打造国际通航大会、全国自驾游大会、丹霞国际摄影节、山丹皇家马场马文化旅游艺术节、全国油菜花节、裕固族民俗文化旅游节等品牌节会赛事，积极推进河西五市宣传营销区域联动， 共推河西走廊精品旅游线路，共同开发客源市场， 持续扩大张掖旅游对外影响力。建设和改造提升一批特色餐饮购物街区，培育壮大乡村旅游、医养旅游、研学旅游、户外运动等旅游业态，打造具有地方特色的精品剧目，丰富旅游产品供给。 建成张掖游客集散中心和导游服务中心，开通连接主要景区的旅游专线，扎实推进“旅游厕所革命新三年行动计划”，补齐旅游服务设施短板。 建成张掖旅游大数据中心和智慧旅游服务、管理、营销平台，提升 旅游信息化服务水平。 探索建立旅游综合执法监管机制 ，持续开展旅游业专项整治行动， 着力打造旅游市场秩序的“首善之区” 。 力争全年接待游客3100万人次以上，实现旅游综合收入200亿元以上。大力发展 金融保险、健 康养老 、咨询评估 等新兴服务业，培育新的经济增长点。 培育壮大商贸流通业增长主体， 加快甘肃嘉禾蔬菜果品冷链物流配送中心、张掖传化公路港物流园等商流基础设施项目建设，完善绿洲农副产品综合批发市场、张掖玉米种子暨农产品交易中心服务功能。进一步健全县乡村三级电商服务体系，确保山丹、临泽 国家电子商务进农村综合示范县通过验收 。

（三）求真务实抓改革，增强区域发展动力

贯彻落实党的十九大对全面深化改革作出的新部署、提出的新要求，找准突破口，抓住关键点，突出实效性，下足真功夫，为破解难题、加快发展注入源头活水，增强持久动力。

持续深化行政审批制度改革。 紧紧围绕“接得住、管得好”，深化“放管服”改革，加强事中事后监管，实现“双随机一公开”监管全覆盖。全面实施市场准入负面清单制度，开展“减证便民”专项行动，充分激发各类市场主体活力。深化环评审批制度改革，开展“规划环评+环境标准”试点，创新“三同时”管理。大力推进网上行权，打造网上办事并联审批平台、快捷高效便民服务平台、信息公开阳光政务平台。进一步清理规范涉企经营服务性收费，持续减轻企业负担。

全面推进农村“三变”改革。 加快农村集体产权制度改革步伐，清理集体资金资产资源，核实“三资”底数，明晰产权关系。严守政策底线，尊重各方意愿，以新型农业经营主体为纽带，以股份合作为主要方式，以实现共同富裕为目标，积极推进“资源变资产、资金变股金、农民变股东”，以“三变”改革带动农村集体产权制度、经营制度改革，进一步激活农村要素资源。探索资金折股量化模式，实现农民、集体和企业互利共赢。

切实抓好重点领域改革。 积极推进国资国企改革、供销社综合改革和事业单位分类改革，统筹推进科技、商事制度、司法、盐业等领域改革，加速释放改革活力。进一步强化市县政府投融资平台作用，扩大投融资规模，支持重大项目建设、重点产业发展。引导支持金融机构创新产品，加大信贷投放力度，提升金融服务实体经济发展质量。积极培育资本市场，推动企业主攻“新三板”，支持更多小微企业通过股权交易直接融资。强化地方政府性债务管理，完善金融监管体系，严厉打击非法集资、金融诈骗，优化金融生态，有效防范化解金融风险。

充分发挥“双创”带动作用。 跟进落实各项“双创”奖补政策，健全完善服务保障体系，确保“双创城市示范”创建通过国家检查验收。持续提升基地服务功能和运营水平，努力建设全国小微企业“双创”特色示范区。着力创新人才发展机制，激发和保护企业家精神，弘扬劳模精神和工匠精神。壮大企业创新主体，实施院企合作项目10项以上，新培育高新技术企业5户以上，新转化各类科技成果50项以上，技术合同交易额达到17亿元以上。

（四）坚持不懈抓生态，不断提升全域生态环境质量

牢固树立和践行绿水青山就是金山银山的理念，坚持“生态优先、保护第一”， 全面推进祁连山生态系统重建，建立山水林田湖草系统保护综合治理新机制，不断 扩大生态比较优势，为筑牢国家西部生态安全屏障作出积极贡献。

全面完成整改整治任务。 集中力量攻坚，全面彻底完成祁连山生态环境问题现场整治任务，深入排查整治保护区外围地带生态环境问题。对各类整治项目进行“回头看”，着力巩固提升整治效果。坚持分类施策，探索补偿机制，依法有序退出保护区内矿产资源开发项目，规范管理旅游和水利水电项目，恢复提升地表植被，积极稳妥处置遗留问题。努力做好保护区搬迁农牧民后续产业培育工作，确保其生活水平不断提高。 持续推进黑河湿地生态环境问题排查整改，加快推进保护区范围和功能区划调整，提高生态环境保护建设整体水平。

加快实施重点生态项目。 全力推进祁连山黑河流域山水林田湖草生态保护修复项目，实施好祁连山水源涵养区浅山区植被恢复与保护、生物多样性保护、土地整治与污染修复等51项重点项目，完成投资25亿元以上。全面加强全域生态文明建设，认真组织实施祁连山生态保护与建设综合治理规划、三北防护林、天然林保护、新一轮退耕还林还草、黑河湿地湖泊治理与保护、草原生态奖补等重大生态项目。统筹推进“一园三带”生态示范工程建设，完成甘州城区段黑河沿岸防护林一期工程，继续推进大规模国土绿化，完成营造林43万亩以上，持续改善走廊生态。加强水生态环境治理保护，完成市区老城区八大明渠水系联通工程，开工建设黑河城区段生态治理二期、大沙河流域等5项河道水系生态治理工程，实施好民乐洪水河、山丹马营河、肃南隆畅河等6项小流域生态治理工程。

统筹治理全域生态环境。 持续深化大气污染防治行动，强化重点行业脱硫脱硝和燃煤锅炉、道路扬尘、机动车尾气、 噪声源控制 等治理，积极推广炉烟炕烟治理新技术，巩固提升 大气环境质量 。进一步强化“河长制”，建立完善“湖长制”“渠长制”， 以市级河流为重点，全面落实水资源保护、水污染防治、水生态修复等措施，维护河湖生命健康。开展土壤污染防治，规范有序实施一批污染土壤治理与修复试点项目。开展农村环境污染第三方治理试点，推进固体废物规范化处理处置，加强农作物秸秆、养殖业粪污、农膜综合治理利用，持续优化城乡环境。 深入推进全域无垃圾示范区创建工作，完善垃圾收运处理体系，力争90%的村庄垃圾得到治理。建立政府主导、企业主体、社会参与的运行机制，实现甘州、山丹、临泽、民乐、高台大部分生活垃圾收集运至垃圾焚烧发电厂集中处理。

建立健全长效保护机制。 加快祁连山、黑河湿地自然保护区分级管理体制和综合管理机制建设，健全完善综合执法、部门联动协调、考核问责奖惩等机制，建设生态环境监测网络管理平台和水生态监控保护信息系统，全面提升管护能力和水平。积极推进祁连山国家公 园体制试点，配合完成资源权属确认、登记、管理机构组建等重点任务。完善生态文明建设评价考核办法，开展领导干部自然资源资产离任审计，坚决守牢生态功能保障基线、环境质量安全底线、自然资源利用上线三大红线。

（五）扬长补短抓民生，加快全面建成小康进程

在全面落实普惠政策、巩固提升社会保障水平的同时，紧盯民生保障短板弱项，持续加大财政投入，回应群众关切期盼，着力解决群众困难 ， 使人民群众获得感、幸福感、安全感更加充实、更有保障。

加快社会事业发展。 深化义务教育城乡一体化发展，推动学前教育普惠发展、普通高中教育多样化发展。有序推进培黎国际职业学院建设，着力提升职业教育办学水平。进一步改善医疗卫生服务条件，市第二人民医院建成投用，完成甘州区人民医院住院部大楼、临泽县人民医院急诊楼等项目年度建设任务。加快“健康张掖”建设， 强化疾病预防控制体系建设 ，完善分级诊疗政策措施，不断提升基本公共卫生服务均等化水平，努力为群众提供全方位全周期健康服务。强化计划生育服务管理，提升计划生育家庭发展能力。巩固提升国家公共文化服务体系示范区创建成果，全力推进国家扩大文化消费试点城市建设，确保市文化馆、丝绸之路文化艺术中心及各县新建公共文化设施建成投用，加快建设市广电传媒中心和非物质文化遗产保护传承中心，新建城市社区文化中心14个、博物馆10个。支持文艺创作，打造一批演艺、戏曲、影视精品力作。扎实推进全国体育产业联系点建设，加快建设全民健身路径工程、户外运动基地等体育惠民工程，促进全民健身和全民健康深度融合。

健全社会保障体系。 坚持就业优先战略和积极就业政策，大力实施 就业创业“三大计划”，城镇新增就业2.4万人以上，城镇登记失业率控制在4%以内，高校毕业生就业率保持在85%以上。稳步推进城镇职工、城乡居民基本养老保险制度改革，建立全市统一的城乡居民基本医疗保险制度和大病保险制度。加快推进老龄事业和产业发展，提高甘州全国综合养老示范基地营运管理水平，建设以居家养老为基础、社区为依托、机构为补充、医养结合的养老服务体系。健全完善城乡社会救助体系，做好留守儿童、空巢老人等弱势群体关爱服务工作，新建城乡社区日间照料中心45个，加快建设市社会福利院和救助管理站等项目。深入实施妇女儿童发展规划，高度重视残疾人事业发展。坚持应改尽改、能改全改，改造棚户区16129套（户），发放住房租赁补贴4800户。建立住房租赁交易服务平台，扩大公共租赁住房保障范围。

加快脱贫攻坚进程。 以贫困村为重点，以贫困户为基本单元，深入推进精细精确精微“绣花式”扶贫，确保精准识别、精准帮扶、精准脱贫。加快推进易地扶贫搬迁， 开工建设278户824人安置住宅。 切实抓好贫困家庭劳动力技能培训，用足用好产业扶贫专项贷款，培育壮大食用菌、草畜、小杂粮等富民产业，确保搬得出、稳得住、能致富。加快贫困片区医疗卫生、健康教育和服务保障体系建设，着力解决因病致贫、因病返贫问题。 全市再减少贫困人口2850人，贫困发生率下降到1.24%。

提升社会治理水平 。 全面推进安全生产领域改革发展，进一步完善落实安全生产责任制，构建安全风险分级管控和隐患排查治理双重预防机制，持续深化重点行业领域专项整治，确保全市安全生产形势持续稳定向好。全力防控和化解食品药品安全风险，保障人民群众饮食用药安全。加大市场监管力度，努力稳定物价。健全应急体系和物资储备管理机制，提高防灾减灾救灾和应急管理科学化水平。严格落实属地监管责任，健全完善保障农民工工资考核问责长效机制，维护农民工合法权益，构建和谐劳动关系。持续治理城市交通拥堵、停车难等问题，不断提升城市精细化管理水平。加强和完善社区治理，强化社会组织作用，提升社会工作水平。拓展基层网格化服务管理，健全自治、法治、德治相结合的基层治理体系。大力开展文明素质教育活动，培育市民意识，提高城市文明程度。深化双拥共建，推动军民深度融合发展。全面贯彻党的民族政策，促进各民族共同团结奋斗、共同繁荣发展。加强宗教事务管理，提高宗教工作法治化水平。深入开展“七五”普法，进一步完善公共法律服务体系。健全信访工作机制，依法规范信访秩序。深入推进“平安张掖”建设，强化治安源头管控，加快建设“雪亮工程”，严厉打击吸毒贩毒、网络欺诈、涉黑涉恶等各类违法犯罪活动，不断提升人民群众安全感。

（六）持之以恒抓作风，提升政府工作效能和水平

牢固树立以人民为中心的执政理念，进一步强化“四个意识”，扭住作风建设这个关键环节，扎实深入开展“不忘初心、牢记使命”主题教育活动，致力建设廉洁、高效、务实的服务型政府，锻造忠诚干净担当的干部队伍。

依法全面履行职责。 全面推进“法治张掖”建设，把法治精神、法治思维、法治方式融入政府工作全过程，强化法治政府建设措施办法，提升依法行政水平。 健全行政决策机制，实施重大行政决策终身责任追究和责任倒查，确保决策制度科学、程序正当、过程公开、责任明确。 进一步 完善政府权力清单、责任清单，全面推进机构、职能、权限、程序、责任制度化，加快建设职能科学、权责法定、执法严明、公开公正、廉洁高效、守法诚信的法治政府。自觉接受人大法律监督、工作监督及政协民主监督和民主党派、无党派人士民主监督，提高人大代表建议和政协提案办理质量。坚持以公开为原则、不公开为例外，及时全面公开政府重大事项和重点工作，完善重大行政决策公众参与制度，保障群众知情权、参与权、表达权和监督权。

驰而不息正风肃纪。 坚持问题导向，深化“三纠三促”，把效果作为检验干部作风的标尺，把问责倒逼与容错纠错结合起来，着力克服当前干部队伍中有困难左绕右拐、有责任上推下卸，担当不足、消极应付的不良风气。严格公务员队伍管理，强化平时考核，严厉整肃庸政懒政怠政行为，坚决纠正“门好进、脸好看、事难办”不作为现象，严防“四风”问题以隐形变异的方式反弹回潮。坚持把雷厉风行狠抓落实作为转变作风的关键，以钉钉子精神做实做细做好各项工作，努力把各项政策措施转化为落地实施的项目、转化为经济发展的质量和效益。

始终保持廉洁本色。 认真贯彻全面从严治党要求，坚决执行“两准则四条例”，切实履行主体责任，坚决把纪律和规矩挺在前面。扎紧制度笼子，完善财政资金支出、工程项目招投标、国有资产管理等制度，加大审计监督力度，从源头上预防和遏制腐败。坚持无禁区、全覆盖、零容忍，盯紧征地拆迁、扶贫攻坚、惠民利民等重点领域，有腐必惩、有贪必肃，切实做到政治清明、政府清廉、干部清正。

各位代表，新时代的张掖发展空间巨大、发展机遇空前，130万人民对过上更加美好幸福的生活充满期待、充满信心。让我们在习近平新时代中国特色社会主义思想指引下，在省委、省政府和市委的坚强领导下，不忘初心、牢记使命，坚定信心、锐意进取， 为决胜全面建成小康社会、建设幸福美好金张掖而努力奋斗！
